# “I feel good because I have saved their lives”: Acceptability of assisted partner services among female index clients and male sexual partners in Kenya

**DOI:** 10.1371/journal.pgph.0001842

**Published:** 2023-05-24

**Authors:** Brienna Naughton, Mercy Owuor, Beatrice Wamuti, David A. Katz, Monisha Sharma, Wenjia Liu, Harison Lagat, Edward Kariithi, Mary Mugambi, Rose Bosire, Sarah Masyuko, Carey Farquhar, Bryan J. Weiner

**Affiliations:** 1 Department of Global Health, University of Washington, Seattle, Washington, United States of America; 2 PATH-Kenya, Kisumu, Kenya; 3 Harvard T.H. Chan School of Public Health, Boston, Massachusetts, United States of America; 4 Department of Child, Family & Population Health Nursing, University of Washington, Seattle, Washington, United States of America; 5 Ministry of Health, Nairobi, Kenya; 6 Kenya Medical Research Institute, Nairobi, Kenya; 7 Department of Medicine, University of Washington, Seattle, Washington, United States of America; 8 Department of Epidemiology, University of Washington, Seattle, Washington, United States of America; University of Washington School of Public Health, UNITED STATES

## Abstract

**Introduction:**

Assisted partner services (APS), or notification for sexual partners of people diagnosed with HIV, is an efficient, effective, and high yield strategy to identify people living with HIV and is recommended by the World Health Organization (WHO). However, there remains a need to further understand the acceptability of APS qualitatively from a client lens, particularly when APS is integrated into the national health system. We investigated acceptability of APS when integrated into HIV services in Kenya.

**Methods:**

Starting in May 2018, APS was implemented in 31 health facilities in Kisumu and Homa Bay counties in western Kenya. From January to December 2019, we conducted in-depth interviews (IDIs) with female index clients (n = 16) and male sexual partners (n = 17) in 10 facilities participating in an APS scale up study. Interviews assessed APS satisfaction, perceived benefits of the intervention, and challenges that may affect delivery or uptake. We applied the Theoretical Framework of Acceptability by Sekhon *et al*. (2017) as a guide to organize our findings.

**Results:**

We find that views of APS are often guided by an individual’s trust in the intervention’s design and implementation, and an interest to preserve one’s health and that of one’s family and children. There were strong and consistent acceptable views of APS as “doing good” and “saving a life” and as a means of showing love towards one’s partner(s). The initial acceptability framing of individuals engaging with APS was predicated either on a feeling of comfort with the intervention, or a wariness of divulging sex partner personal information. Health care workers (HCWs) were seen to play an important role in mitigating participant fears linked with the intervention, particularly around the sensitive nature of HIV disclosure and sexual partners. Clients noted considerable challenges that affected acceptability, including the risk to the relationship of disclosing one’s HIV status, and the risk of intimate partner violence.

**Discussion:**

We found that APS is acceptable as a strategy to reach male sexual partners of females diagnosed with HIV, and these findings provide opportunities to inform recommendations for further scale-up. Opportunities such as focusing on intervention confidentiality and appropriate counseling, excluding female clients at risk of IPV from this intervention, and highlighting the altruistic benefits of APS to potential clients. Understanding the perspectives of clients receiving APS in a real-world setting may be valuable to policy-makers and stakeholders interested in scaling up or enhancing APS within health systems.

## Introduction

Globally, an estimated 6.1 million people living with HIV do not know their HIV status [1]. In sub-Saharan Africa (SSA), which continues to be disproportionately affected by HIV as a cause of morbidity and mortality [2], people living with HIV (PLHIV) are generally diagnosed through facility-based HIV testing. However, testing coverage has been insufficient to curb the epidemic, particularly among men and key populations (such as sex workers and men who have sex with men) [3]. Reaching undiagnosed individuals, getting them tested, and linked to care is critical to achieving the UNAIDS 95-95-95 objectives by 2025 [4]. Assisted partner services (APS) is an important public health strategy aimed to prevent the spread of sexually transmitted infections, including HIV, by identifying, testing, and treating sexual partners of individuals diagnosed with HIV [5]. The process generally includes a health worker eliciting partners from consenting individuals testing positive for HIV (index clients), then assisting the client with notifying their partners [5].

Randomized controlled trials and demonstration projects in SSA have found APS to be safe and effective in testing potentially HIV-exposed partners, and in finding high HIV positivity (30–63%) and high cluster of differentiation 4 (CD4) median counts among partners, indicating that individuals are identified earlier in their disease course compared to clinic testing [6–11]. In 2016, the World Health Organization (WHO) released testing guidelines recommending offering APS as part of a comprehensive package of testing and care to all PLHIV [5]. APS is increasingly being implemented across SSA. Kenya guidelines for HIV testing now recommend APS implementation as part of routine HIV testing services (HTS), but there are still challenges for the implementation and scale-up of APS nationwide, including with human and financial resources, such as the effort and funding needed to trace and test partners [12]. In general, APS are under-utilized in the country, and a need exists to further understand the coverage and implementation gaps [11].

Acceptability of healthcare interventions is an important measure of the perception among stakeholders that a given treatment, service, practice, or innovation is agreeable, palatable, or satisfactory [13]. Acceptability of APS has primarily been assessed in controlled settings such as APS trials and demonstration projects, and has often been assessed quantitatively by proxy measures [8, 11, 14–23]. In these controlled studies, APS has been found generally acceptable, with high acceptance rates in Kenya, including with 67% uptake in a cluster-randomized controlled trial (RCT) [8] and 89% in a cross-sectional facility-based study undertaken in five health facilities [11]. However, there remains a need to further understand the acceptability of APS qualitatively from a client lens, particularly when APS is integrated into the national health system.

In Kenya, approximately 8% of PLHIV do not know their status [24]. In this qualitative study, we aimed to investigate APS as an intervention to increase awareness of HIV status, and we explored the acceptability of integrated APS from the perspective of female index clients (FIC) and male sexual partners (MP) receiving APS in Homa Bay and Kisumu Counties, two Kenyan counties with the highest HIV prevalence in the country (25.7% and 19.3% respectively [24]). To guide our findings, we leveraged the Theoretical Framework of Acceptability by Sekhon et al. (2017), a framework designed to add rigor and robustness to assessing the acceptability of complex healthcare interventions [25]. In utilizing this framework, we aim to present an investigation of acceptability of integrated APS in SSA and suggest opportunities to strengthen scale-up of integrated APS.

## Methods

### Study overview

This study was conducted within the APS scale-up study, a hybrid type II implementation science study [26] in Homa Bay and Kisumu counties, between May 2018 to March 2021. The study team consisted of a collaboration between the University of Washington, PATH (a non-governmental organization), and the Ministry of Health in Kenya. The study aimed to determine the effectiveness of APS when integrated into existing routine HIV testing, prevention, and care programs, and to evaluate the implementation of APS in these settings, including the integration, implementation fidelity, acceptability, demand, and costs of the intervention. This analysis investigated the acceptability of APS from the perspective of FICs and MPs.

### Study sites, participants

From January to December 2019, we conducted in-depth interviews (IDIs) in 8 facilities participating in the APS scale-up project. Facilities were chosen to maximally represent a range of facility types, including both APS high-performing and low-performing facilities (performance defined as the number of MPs enrolled per FIC) and geographic location/size including a range of facility levels from small rural outposts to high-volume urban clinics.

From each facility, we purposively selected one FIC who named ≤2 MPs, and one FIC who named >2 MPs, for a total of 16 FIC IDIs. Eligibility for FICs in this analysis included being enrolled in the APS scale-up project. Two MPs were recruited from each facility (three from one facility), for a total of 17 MP IDIs. Inclusion criteria for FICs included testing positive for HIV, having been enrolled in APS for six weeks, and not having reported intimate partner violence after receiving APS. Inclusion criteria for MPs included testing positive for HIV and eliciting at least one female partner. There were no exclusion criteria.

### Study procedures and data collection

FICs and MPs at these facilities who enrolled in APS were invited to participate in phone IDIs with a qualified qualitative interviewer (MO). Those interested were screened for eligibility and provided oral and written informed consent. The IDIs were conducted in Kiswahili, Luo, or English, based on the language preference of the client. The recorded interviews were transcribed verbatim and translated into English for analysis by our study team. The interviews took place at a study facility or another safe and private location chosen by the participant. The interviewer used a semi-structured interview guide, which addressed APS satisfaction, perceived benefits of the intervention, and challenges that may affect APS delivery or uptake. The interview guides for FICs and MPs were similar; the male guide included perspectives on how males felt being contacted by a routine HTS provider and being informed that they were exposed to HIV. Interviews were audio-recorded and transcribed verbatim.

### Data analysis

An inductive thematic analysis [27] was conducted to identify emerging themes. After reading the transcripts multiple times, two researchers (BN and MO) independently openly-coded the data line by line, and then reached a consensus on the codebook through discussion with the study team. After the codebook was tested with two transcripts, the two researchers coded all the transcripts independently and discussed findings with each other and the study team. Through group discussion and iteration, similar codes were aggregated to form categories based on similarity/overlap, and BN and MO evaluated the categories to ensure the codes were appropriately aggregated with additional reviews of the interview texts to ensure codes and categories did not deviate from the participants’ meaning. The categories were then further classified as themes and subthemes based on group discussion and alignment. Themes that emerged from the data were identified and discussed. The entire interview for each participant was the unit of analysis.

We then applied the theoretical framework of acceptability (TFA) proposed by Sekhon *et al*. [25] to guide our presentation of findings. The TFA was developed as a multi-construct theoretical framework of acceptability of healthcare interventions that can be applied to assess acceptability from the perspective of both intervention deliverers and recipients [25]. This framework has been utilized in a number of complex health intervention studies [28–30], and leveraged regionally in SSA [31–33]. Final themes were mapped onto six of the dimensions of the TFA: affective attitude, burden, effectiveness, self-efficacy, ethicality, and intervention coherence. In the analysis, it emerged that participants did not distinguish opportunity costs from burdens or perceived effectiveness; therefore, we did not retain the construct in our analysis. Data were analyzed in ATLAS.ti version 8.4.4, and the findings of this study are reported according to the consolidated criteria for reporting qualitative studies (COREQ) [34].

### Ethical consideration

The study was approved by the Kenyatta National Hospital Ethics and Research Committee (P465/052017) and the University of Washington Institutional Review Board (STUDY00002420). All participants provided written informed consent. Girls who are ≥15 years old are considered emancipated minors in Kenya if they meet one of the following criteria: married, pregnant or have had a sexually transmitted infection, including HIV. It was under this condition that those ≥15 years were enrolled. Each recorded interview and corresponding transcript was assigned an ID number and stored in an encrypted place with no names or identifying information included. The recordings will be destroyed at the latest 5 years after the study completed.

## Results

We conducted 33 IDIs, 16 with FICs and 17 with MPs with approximately equal representation from Homa Bay and Kisumu counties. FICs were between 15–36 years old, and MPs ages ranged from 23–52 years old (Table 1). Most FICs (75%) and MPs (94%) had completed at least primary school.

**Table 1:** Characteristics of the participants (n = 33).

Characteristics	Female index participants (n = 16) N (%)	Male sexual partners (n = 17) N (%)
**Age** (years)	15–36	23–52
**Study site**		
Kisumu	8 (50%)	9 (53%)
Homa Bay	8 (50%)	8 (47%)
**Level of education completed**		
Some primary school (started, not completed)	4 (25%)	1 (6%)
Completed primary school	6 (37%)	5 (29%)
Some secondary education (started, not completed)	2 (13%)	3 (18%)
Completed secondary education	3 (19%)	7 (41%)
Post-secondary education	1 (6%)	1 (6%)

**Fig 1:**
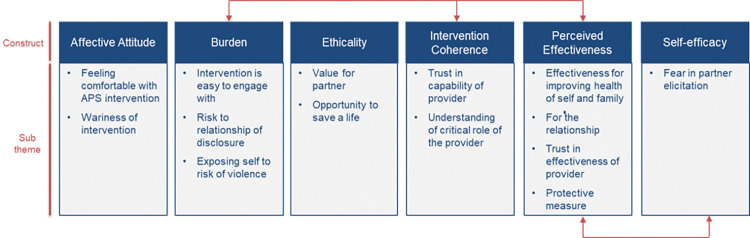
APS acceptability framework, adapted from Sekhon et al. **[25].** Note: The red arrows reflect evidence of linkage between the constructs found in the analysis, with responses from FICs and MPs describing similarities and/or interdependencies amongst specific constructs in their perceptions of acceptability.

The findings present the acceptability of APS from the participant perspective, adapted from the TFA. The framework was adapted to better align to the APS context, including with refined constructs and visual evidence of linkages between constructs, particularly between burden, intervention coherence, and perceived effectiveness, as well as between perceived effectiveness and self-efficacy (Fig 1).

### Affective attitude

Affective attitude is defined as how an individual feels about an intervention [25]. Participants expressed a wide range of feelings regarding the APS intervention, from comfort with to wariness of APS. For participants conveying comfort with the intervention, the supportive roles of the health care workers (HCWs) in both counseling and notifying sexual partners were highlighted as important facilitators.

*Interviewer*: *Did you have fears or you were just comfortable to give their contacts*? *Participant*: *I did not have any fear and that is why I was giving out their contact*. *Interviewer*: *You were just comfortable*, *why were you comfortable*? *Participant*: *Because I had been counselled and taught and so I also wanted to help others as well*. *(IDI 036*, *FIC*)

*Because of the approach they [HCWs] gave me*, *they were open with me and I was also open with them and didn’t feel uncomfortable about anything*. *(IDI 035*, *MP*)

Notable discomfort and wariness of naming and notifying partners was expressed as well. Some participants explained that “there are some private matters that feel awkward when someone asks you about and is not easy to speak up,” and reflected they were thinking to themselves “how do I start explaining such a thing to someone else.” The role that the healthcare workers played was seen as mitigating these fears and wariness, but this challenge was reflected consistently.

*Although when she [HCW] first mentioned it to me*, *I was worried how I was going to approach my sexual partners but then she told me I should leave that to her because that is now her job*. *(KII 038*, *MP*)

### Burden

Burden refers to the perceived amount of effort that is required to participate in an intervention [25]. The sub-themes identified included the technical ease of engaging with the intervention, but also the risk to the relationship of disclosing one’s status, and the risk of a woman being exposed to interpersonal violence.

The low burden of engaging with the intervention was noted routinely as “easy” and “without any fear,” particularly with the benefit of “helping me and my partners,” and one’s children. However, the consistent detracting burden noted for APS acceptability was the risk and fear of repercussions of having one’s status disclosed. Participants explained that partners “might leave them” if partners find out about their HIV status during the APS process. MPs relayed concern about the risk of disclosing multiple sexual partners, particularly in exposing those non-primary partners with whom they are in “secret” relationships.

*The difficulty was the part where I was expected to expose my sexual partners*. *This is someone we have been having sex with in secret and now I was about to expose her and tell this provider who they are and the things we have done together*. *I was scared that she could find out that I was the one who shared their details with the provider*. *However*, *because of the kind of teachings I got from the provider*, *I opened up because I wanted all of us to get help*. *(IDI 055*, *MP*)

FICs expressed similar sentiments, but often with the elevated risk of their MPs “leaving them,” dissolution of their relationships, and often the corresponding loss of access to financial resources and housing. These burdens of APS escalated to the risks of intimate partner violence (IPV) against the FICs if the sexual partners could somehow find out about having been named to healthcare staff.

*Sometimes they think they can be tracked and the husband will find out that they are the ones who gave out the contact*. *And if they find out then sometimes they can be beaten thoroughly*. *You can even be sent away from the home*. *(IDI 061*, *FIC*)

MPs also highlighted IPV as a concern that their female partners, or any FIC, would be considering in the acceptability of APS, in that any resulting disclosure of HIV status would be dangerous.

*The reason why the women will fear is that–you know some of us men are very arrogant and some of the men when they will hear about this then they will not take it lightly*. *They will be very harsh and they can even become violent and the woman can be beaten (IDI 064*, *MP*)

### Ethicality

According to the TFA, ethicality is the extent to which the intervention is a good fit with an individual’s value system [25]. Interestingly, though the interview questions did not explicitly ask about value systems or ethics of this intervention, two sub-themes were repeated in the IDIs: value for the partner, and the opportunity for this intervention to save lives. Participants expressed that only those who “didn’t value their relationships” or “didn’t love each other” would not accept APS, and that if they truly “loved someone you wouldn’t want to harm them” by not naming partners to be notified.

Participants suggested that their perspectives on the acceptability of APS were influenced by strong motivations to “do good” and “save a life,” and this was noted across male and female interviews.

*I feel good because I have saved their lives somehow by helping them get tested and enrolled into care*. *I have saved their lives and now each one of us can now take good care of their lives and enroll into care*. *(IDI 048*, *FIC*)

This facilitating factor i.e., “to help their partners and family,” was consistently identified as playing a motivating role for the intervention’s acceptability.

*I didn’t find it bad because I wanted to help my life and that of the other partners as well*. *I wanted them to be reached and talked to so that if they also find it good they can also enroll into care*. *(IDI 067*, *FIC*)

### Intervention coherence

The next theme in Sekhon *et al*.’s framework is intervention coherence, which denotes the extent to which the participant understands the intervention and how it works [25]. In our study, we found that aspects regarding the acceptability of APS were not only expressed in terms of ‘understanding’ the intervention and how it works, but also in awareness of and understanding in the role of trust in the intervention design. Participants consistently indicated that the acceptability of APS was predicated on trusting “the provider not to reveal their identity to their sexual partners,” as there was a prevailing risk that “the provider can easily mention their names,” but noting that “they are not allowed to do that”.

*The reason some people would refuse to give the contact details*, *let me start from there*, *is because she/he knows that at the end of the day the partner would want to know who gave out his/her contacts and will go back to her/him and ask why they gave out their contacts*. *That is why some people don’t like doing that*. *(IDI 035*, *MP*)

Finally, FICs and MPs explained that an important component of APS was in understanding the design of the intervention, with a HCW playing a critical role in counseling and reaching out to partners instead of the participant having to do it themselves. FICs explained how their MPs were more willing to listen to a HCW than their female partners, where “if he hears it from someone else then he might take it seriously,” and “it is better if the provider just contacts them and notifies them.” Both female and male participants “found it hard to go and talk to them [sexual partners] myself,” and that APS was effective in doing so instead.

### Perceived effectiveness

Sekhon *et al*. defines the perceived effectiveness construct as the extent to which an intervention is expected to achieve its purpose from the participant’s perspective [25]. Participants distinguished their framing of effectiveness in the context of acceptability across several sub-themes, including effectiveness of improving health, of impact on their relationship with their partner, and the importance to the success of the intervention which was the role of the HCW eliciting and notifying partners.

Participants expressed favorable benefits of participating in APS in that they understood their own HIV status, and that of their partner. Participants felt that they had more information about themselves due to APS, and one FIC explained that she is “aware of many things which I didn’t know before,” in regard to her own status. Though the APS intervention is designed for confidentiality, participants felt that engaging in the intervention supported their male sexual partners in finding out their own statuses, which ultimately could protect the health of the FIC. One FIC explained why the effectiveness of APS was important, in that “I wanted [my partners] to be reached and talked to so that if they also find it good they can also enroll into care.”

Though FICs expressed the motivating role of protecting partners and family more than did MPs, some MPs expressed similar urgency to engage with the intervention from the lens of perceived effectiveness.

*You see I was considering that if I kept quiet then I would have died and my children would remain alone without a father*. *So I got courage and decided that let me just be open up so that both of us could be helped*. *(IDI 050*, *MP*)

Participants also explained how APS supported their relationships by facilitating more openness and communication by leveraging provider-initiated contact for HIV testing.

*So if I give out his contact information in secret and then he is traced and found that he is also on care then that would make us to be open to each other*. *(IDI 061, FIC)*

*This experience was a good one because at least it saved us from the chaos that would have erupted if I was the one who went home and told my wife about my status*. *When you go home and tell your wife about it*, *she will be shocked and it will create problems in the house and cause a lot of blame games on who brought it to the other*. *However*, *because the results came back when we were both together*, *we went back and talked about it and sorted everything out amicably without causing chaos or problems in our marriage*. *(IDI 055*, *MP*)

Trust in the confidentiality of the intervention was thus important, but there were also reflections that participants, particularly male participants, trusted the HCWs to be more successful in ensuring linkage to care of their partners than they could on their own.

*The reason I chose her to do it on my behalf is because she is more knowledgeable on how to handle this issues than myself*. *She really challenged me and I trusted her to do a better job getting my wife and sexual partners on my behalf*, *counsel*, *test*, *and enroll them into care*. *So this is the reason why I decided to share their contacts with the provider because I don’t have the experience of counselling anyone…That is why I chose her to go and approach my sexual partners to handle the difficult parts*. *(IDI 055*, *MP*)

MPs additionally critically noted that APS was effective in reaching them for HIV testing, and that they otherwise would not have known their status.

*I did not know my HIV status but being that I was called and got tested made me know my status*. *I can say that it has really helped me up to now because I am still alive and I am continuing with my business*. *If it were not for the program*, *I would have died a longtime ago*. *(IDI 049*, *MP*)

Finally, some FICs also explained how this risk of IPV motivated the engagement with APS and framed its perceived effectiveness, in that the role of the assisted notification served as a potential protective measure for themselves.

*Interviewer*: *So it is fear that makes most women chose provider referral*. *Participant*: *Yes*, *it is mostly because of fear*. *Women would think about how their spouse or partner usually reacts to things and say they cannot dare face him*. *Some of them change when things happen and they become very wild like a lion or burn like fire and are very mad*. *So they will just think that if this guy reacts very badly to simple things*, *will he not kill me when he hears this*? *(IDI 067*, *FIC*)

### Self-efficacy

The self-efficacy construct is defined as the participant’s confidence that they can perform the behaviors required to participate in the intervention [25]. This links closely with the perceived effectiveness expressed above, with respondents framing their ability to engage with the intervention often in the context of how well the intervention would work in preserving confidentiality and in helping oneself and one’s partner. Participants noted that their initial hesitation and lack of confidence in the intervention’s requirement to name partners affected their views of the acceptability of APS.

*It was not easy for me to make that decision because I felt she would breach the confidentiality and disclose my status to my sexual partners*. *But with time*, *after I saw her experience and the way she managed to counsel me and convince me to get tested and enroll into care*, *I then said let me just let her reach out to my sexual partners because I felt she was the only one who could help me with it successfully*. *(IDI 055*, *MP*)

While lack of confidence was noted throughout, the primary sub-theme for self-efficacy was the more specific fear of partner elicitation, due to barriers including stigma and the burden and risks of disclosure, including relationship dissolution and violence. Some participants felt a real risk in engaging with APS across these components, and this impacted their confidence in engaging with the intervention.

*When you have someone you want to get married to*, *you will start thinking*, *‘What if he is invited to the hospital and he finds out that I am the one who gave out his contacts*. *Will he leave me*?*’ So this is where the fear comes in*. *What will happen to me when he leaves me*? *(IDI 056*, *FIC*)

## Discussion

In this study, we aimed to qualitatively understand the acceptability of APS from participants who choose to engage with APS while it was being integrated into existing routine HIV testing, prevention, and care programs. We found that views of APS are often guided by an individual’s trust in the intervention’s design and implementation, and an interest to preserve one’s health and that of one’s family and children. There was a consistent acceptable view of APS as “doing good” and “saving a life,” and as a means of showing love towards one’s partner(s). These perspectives provide important insight into the perceptions of individuals who accept APS and may inform recommendations for further scale-up of integrated APS.

Our findings in the real-world setting of HIV services are consistent with those obtained in studies nested within randomized controlled trials. For example, qualitative studies nested within two recent APS RCTs in Kenya have identified both challenges with awareness and lack of trust in HCWs, and have similarly identified the importance of appropriate HCW training and sensitization for APS participants [21,22]. Other research has found similar challenges to the intervention, including lack of trust in the health system and gender-specific challenges as inhibitory to partner elicitation [14–20].

Guided by the framing of Sekhon *et al*.’s TFA, we found alignment but also important distinctions that helped to further elucidate acceptability in the context of APS. We found that the affective attitude of individuals engaging with APS was primarily predicated either on a feeling of comfort with the intervention, or a considerable wariness of the personal information required to proceed with naming partners. The importance of the HCWs was critical to shaping the affective attitude of participants, particularly in playing an important role in mitigating fears of the intervention regarding the sensitive nature of HIV disclosure and sexual partners. The criticality of well-trained providers for APS is in line with similar research [18, 21, 35], and will continue to serve an important role as APS programs expand.

We found that acceptance was viewed by the participants from the lens of either a low or a significant burden. There was a low initial burden of engaging with an “easy” intervention, in that simply naming one’s sexual contacts was all that the participant needed to do. However, this did include considerable risks that impacted acceptability, including the risk to the relationship of disclosing one’s status, and the risk of a woman being exposed to intimate partner violence. When understanding the acceptability of APS as it is being scaled up, understanding the perspective of participants who are assessing the intervention from a perspective of its burden could be useful for HCWs to mitigate these fears. While the view of APS as an “easy” intervention is novel, our broader findings also reflect similar research in Kenya and Malawi, with participants expressing discomfort with the risk of disclosure in their relationships, and risk of relationship conflicts [15, 21]. Improvements of APS delivery could include an early targeted counseling session focused the prevention of perceived risks of APS and redirecting of those at risk of IPV towards other related services.

The individual assessments of burden that participants undertake when viewing the acceptability of APS is closely related the importance of intervention coherence, with trust required in the capability of the HCWs, and in understanding the critical role that the HCW plays throughout the intervention. This is also closely associated with the perceived effectiveness of the intervention, specifically the trust required that the HCW can effectively implement the intervention. Respondents often spoke of these constructs in a holistic manner, particularly regarding trust. Mistrust in HCWs and fears about lack of confidentiality have been found to be barriers to APS in other settings as well, including the importance noted in a qualitative study in Kenya of establishing trust between the client and HCW in order to increase uptake of APS [21], and the importance of trust in the HCW to maintain confidentiality in Barbados [36]. The fear of stigma created by broken confidentiality is consistent with those found in other settings including Ethiopia [37], Malawi [38], Tanzania [39], Ghana, [40], and Barbados [36]. Participant trust in the different facets of APS is critical to acceptability and comfort with the intervention, and HCWs play an important role in building and maintaining that trust. As APS is being scaled to new facilities, focus on HCW expertise in delivering a confidential service and in maintaining that trust with participants will need to continue to be prioritized.

The self-efficacy and perceived effectiveness constructs, though distinctly defined, were also related, in that respondents framed their self-efficacy and ability to engage in part based on their perceived effectiveness of the intervention’s components, such as confidentiality and impact. Participants noted that their initial hesitation and lack of confidence in the intervention impacted their views of the acceptability of APS, but when overcome, they were able to distinguish their framing of effectiveness in the context of acceptability across a number of sub-themes, including effectiveness of improving health, of impact on their relationship with their partner, and the importance to the success of the intervention of the role of the HCW eliciting and notifying sexual partners. Importantly, we found that participants explained that an effective component of APS was due to the design of the program, in that a HCW played a critical role in counseling and reaching out to partners instead of the participant having to do it themselves. This is in close alignment with the findings on trust in HCWs as well. This also had an important gender aspect, as FICs found that their view of acceptability of APS was impacted by how their MPs were more willing to listen to a health care worker than to themselves as females (as noted under the Intervention Coherence construct). While APS in Kenya is known to be more effective in reaching males than females, given that a smaller percentage of men in Kenya know their HIV status due to fewer testing opportunities [41], this was a unique finding with regards to MP acceptability of APS compared to partner notification, and may have implications for policies and recommendations. Ultimately, policy-makers and stakeholders will need to support the sustainability of the effectiveness of this intervention as APS scales up, and evidence showing improvements to health, and to relationships, may prove helpful for recruiting additional participants going forward.

The perspectives on ethicality of APS are in line with similar research. The WHO has noted the role that social responsibility plays with APS, with both personal and public health benefits of identifying people living with HIV, linking them to care and treatment, and preventing further HIV transmission [42]. Quinn *et al*. highlighted the altruistic benefits of partner notification in enabling a partner’s awareness of their exposure to HIV and allowing them to know their status and access treatment early [43], and Njozing *et al*. discussed how the motivation to notify one’s sexual partners is influenced by the patients’ ethical responsibility and concern for the partners’ health [44]. Our results also suggest that ethicality affects acceptability of APS. We found that there was a strong and consistent acceptable view of APS “doing good” and “saving a life,” and a means to show love towards one’s partner(s). These findings could prove valuable for helping to frame APS when enrolling participants. Applying these findings in practice may include highlighting the altruistic benefits of APS in community sensitization campaigns, and/or in recruiting individual participants at facilities.

Finally, the aspect of fear is salient throughout the findings, including impacting the affective attitude of participants, but also in framing how participants viewed the burden of APS, the perceived effectiveness, and their own self-efficacy to engage with the intervention. Fear appeared to be widely defined in our findings however, ranging from unease about HIV disclosures, apprehension of one’s willingness to name their partners, to distress and anxiety about partner responses and individual safety. Further research will be important to understand how nuanced framings of fear impact acceptability of the different components of APS, both from the lens of individuals but also of couples themselves.

This study contained several strengths. APS acceptability was evaluated within real-world settings, with the APS intervention integrated into the standard of care at the site facilities. Participant perspectives were directly investigated, as opposed to using proxy measures to infer acceptability. These aspects give a realistic view of the perspectives of acceptability for planning APS scale-up nationwide. Additionally, both male and female perspectives were incorporated, and these perspectives were drawn from facilities with varying participant volumes and APS performance levels. Also, the field of implementation science has historically been under-served by frameworks for acceptability [26], and this study uniquely applies the comparatively new TFA to the APS intervention.

This study had a number of limitations. Importantly, our study only captured individuals who accepted the intervention themselves–this selection bias prevented us from learning about the perspectives of individuals who refused APS, and who may have found APS unacceptable. Previous research within this study population has however investigated reasons for declining APS [23], and the perspectives on acceptability of those who do engage with the intervention do provide important insight for intervention scale-up. Further research should investigate these barriers to accept APS. Secondly, the interview guides were not formed according to the TFA constructs, which may have limited application of the TFA to the data, and/or result in constructs not being fully contextualized. However, by applying the TFA to the data collected, the interview guides were not constrained by a preconceived framework, and may reflect a more comprehensive perspective of acceptability. There is additionally variable, similar, utilization of the TFA in the literature, and studies have used and applied the TFA in different ways, including applying the TFA after data collection, resulting in informative findings [33, 45]. Finally, this study may be limited in generalizability, as this focused exclusively on heterosexual partnerships in western Kenya, and APS acceptability may differ in additional types of partnerships and/or vary regionally.

## Conclusion

These findings provide opportunities to inform recommendations for further scale-up. These include ensuring that APS providers are well-trained to deliver the intervention confidentially and with appropriate counseling, to potentially excluding female participants at risk of IPV from this intervention, and to highlight the altruistic benefits of APS during recruitment of participants. Understanding these perspectives of participants receiving APS in a real-world setting may be valuable to policy-makers and stakeholders interested in scaling up or enhancing APS within health systems.

## Supporting information

S1 Appendix Interview guide–Female index clients. (PDF)

S2 Appendix Interview guide–Male partners. (PDF)
