# Supplementary material for: “I feel good because I have saved their lives”: Acceptability of assisted partner services among female index clients and male sexual partners in Kenya
Source: PLOS Glob Public Health. 2023 May 24;3(5):e0001842. doi: 10.1371/journal.pgph.0001842 (PMC10208474; doi:10.1371/journal.pgph.0001842)
Supplement: S1 Appendix — (PDF) [file pgph.0001842.s001.pdf]

ACCEPTABILITY AND DEMAND INTERVIEW GUIDE – FEMALE INDEX CLIENTS

Instructions:
Find a suitably private place to meet and to conduct the interview. Try to ask all the questions below in the order given, but it is more important to maintain the flow of discussion while covering all the questions. Suggested probes have been included. Start with the following introductory script:

Hi, my name is ____________. Thanks for agreeing to participant in the interview. First, I am wondering which language you are more comfortable to use during the interview? [Wait for the participant to give their preference]. *I would like to record the discussion so we don't miss any important things that you say, but we will not include your names or identifying information in the final typed-up transcripts of the interview, and the tapes will be destroyed at the latest 5 years after the study completed. Is it okay if I record our discussion?* [Wait for the participant to give verbal consent to recording]

Before we start, I would like to let you know that there are no right or wrong answers for the questions. We are interested in knowing your experience and your thoughts about Assisted Partner Notification Service. Your responses will help us understand how to make the Assisted Partner Notification Service better and will in NO way impact any service you will receive. So please feel free to be open and share your point of view. Also, you do not have to answer all the questions. If at any time you do not wish to answer a question or want to stop the interview, just let me know.

Participant ID Number: __________________ Date: _____/_____/________(dd/mm/yyyy)<br>Interviewer: _____________ Language used in the interview: _______________<br>Starting Time: _________ Length of session: _________ minutes<br>Recording device: _____________ Recording digital file #: _______________		
1. Participant's Age:<br>_____________	2. Participant's Gender:<br>▭ female<br>▭ male	3. Place of Residence:<br>▭ Kisumu<br>▭ Homa Bay
4. Name of facility from where the participant received aPNS:__________________	5. Number of partners the participant elicited in aPNS: ______________	
6. Marital Status (at time of interview)<br>▭ single / never married<br>▭ married monogamous<br>▭ married polygamous<br>▭ cohabiting (live-in partner)<br>▭ divorced or separated<br>▭ widow/widower	7. What is the highest education you completed?<br>▭ never attended school<br>▭ some primary school but did not complete<br>▭ completed primary school<br>▭ some secondary school but did not complete<br>▭ completed secondary school<br>▭ post-secondary education<br>Please specify: _______________	

Opening Questions

I will start by asking you questions about your experience of HIV testing.

a. How was the experience of being tested for HIV?
b. How did it feel to learn that the HIV results were positive?

Now I will ask your experience and thoughts about Assisted Partner Notification Services, or aPNS for short.

Acceptability of Assisted Partner Notification Service (aPNS) - Provider Referral (PR)

When you first learned that you have HIV, you were offered and accepted "Assisted Partner Notification Services." You were asked to provide contact information for your sexual partners for the past three years. The HIV testing service (HTS) provider used that information to contact your sexual partners, notify them that they had been exposed to HIV (but kept your identity confidential), and encouraged them to get tested for HIV.

1 Were you aware of the aPNS service before the health care provider offered it to you?
- What did you know about it?
- Where did you learn about it?
- If no, what were your impressions when you received the call from the HTS provider? Did you feel comfortable taking up the service?
- Did you feel that you had enough information about the service to accept to use it?

2 Thinking back, how would you describe your experience receiving Assisted Partner Notification Services? Was it a good experience, a bad experience, or a little bit of both?
- What made it a *[good, bad, mixed]* experience?
- What did you like about receiving Assisted Partner Notification Services? What did you not like about it?

3 What thoughts or feelings did you have when the HTS provider asked if you would like to receive Assisted Partner Notification Services?
- How easy or hard was it to make the decision to receive Assisted Partner Notification Services? What made it [*easy or hard*]?
- How do you feel about this decision now?

4 What was it like giving information to the HTS provider about your sexual partners, including their contact information? What thoughts or feelings did you have?
- How easy or hard was it to give this information to the counselor? What made it [*easy or hard*]?
- Was there any information you did not feel comfortable sharing? What made you feel uncomfortable sharing this information?
- How do you feel now about giving this information to the counselor?
- What are the most important things that influence the decision about whether to give your partners contacts after testing HIV positive or not? Why?

5 Do you know whether the HTS provider contacted your current partner(s)?
- If so, could you describe how your current partner(s) was/were contacted by the counselor?
- What was his/her reaction to being contacted?

- How did you feel about his/her reaction at the time?
- How do you feel about his/her reaction now?

6 Do you know whether the HTS provider contacted your <u>previous partners</u>?
  - If so, how did that go?
  - How did you feel about it at the time?
  - How do you feel about it now?

7 Reflecting on your experience, what <u>benefits</u> did you experience in receiving Assisted Partner Notification Services?

8 Reflecting on your experience, what <u>challenges</u> did you experience, if any, in receiving Assisted Partner Notification Services?
  - Did you experience any costs in receiving Assisted Partner Notification Services? (If so, what were they?)

9 Overall, based on your experience, do you think receiving Assisted Partner Notification Services was a good idea?

10. If you had a friend who tested positive for HIV, would you advise that friend to get Assisted Partner Notification Services?
    - Why/why not?
    - If yes, what would you say to that friend?

Perceived Demand for Assisted Partner Notification Service (aPNS)

Now, I would like to ask you a few questions about what people in your community might think about "Assisted Partner Notification Services." There are no right or wrong answers. I am interested in hearing your opinion.

1 Have most people you know heard about aPNS?
  - From where have people heard about it?
  - Have any come to talk to you specifically about aPNS?
  - What do they say about aPNS?
  - What are their positive reactions to aPNS?
  - What are their negative reactions to aPNS?

2 When you think about the people in this community, how many would want HTS providers to notify their current and past sexual partners if they tested positive for HIV? Would you say most of them, some of them, or few of them?

- What makes you think so?
- Do you think the rest would rather notify their current or past sexual partners themselves without assistance from HTS providers?

3 Do you think there are some specific groups of people would want HTS providers to notify their current or past sexual partners? Which groups?

[Interviewer: probe for the following groups if not mentioned: women, men, young people, or adults]

What makes you think (INSERT GROUP) would want HTS providers to notify their current or past sexual partners?

[Interviewer: probe for the following possible reasons if not mentioned: want their partners to get tested, would prefer assistance informing partners, takes less time]

4 Do you think there are some specific groups of people would not want HTS providers to notify their current or past sexual partners? Which groups?

[Interviewer: probe for the following groups if not mentioned: women, men, young people, or adults]

What makes you think (INSERT GROUP) would not want HTS providers to notify their current or past sexual partners?

[Interviewer: probe for the following possible reasons if not mentioned: do not care if their partners are tested, would rather inform partners themselves, distance to facility, fear of IPV, cost of care]

5 Would you consider championing aPNS in your community?

- If yes, why?
- How would you do it?
- If no, why?

Conclusion

Thank you for your time in sharing your experience with us. We will use this information to make the Assisted Partner Notification Service better as they may become part of HIV prevention care and support services in Kenya. We will keep your information confidential and safe. Thank you very much.

Ending Time: ________________________
